# Alteration of the gut microbiota following SARS-CoV-2 infection correlates with disease severity in hamsters

**DOI:** 10.1080/19490976.2021.2018900

**Published:** 2021-12-29

**Authors:** Valentin Sencio, Arnaud Machelart, Cyril Robil, Nicolas Benech, Eik Hoffmann, Chloé Galbert, Lucie Deryuter, Séverine Heumel, Aline Hantute-Ghesquier, Anne Flourens, Priscille Brodin, Fabrice Infanti, Virgile Richard, Jean Dubuisson, Corinne Grangette, Thierry Sulpice, Isabelle Wolowczuk, Florence Pinet, Vincent Prévot, Sandrine Belouzard, François Briand, Martine Duterque-Coquillaud, Harry Sokol, François Trottein

**Affiliations:** a Univ. Lille, CNRS, Inserm, CHU Lille, Institut Pasteur de Lille, U1019 - UMR 9017 - CIIL - Center for Infection and Immunity of Lille, F-59000 Lille, France; b Centre National de la Recherche Scientifique (CNRS), UMR 9017, F-59000 Lille, France; c Institut National de la Santé et de la Recherche Médicale (Inserm) U1019, F-59000 Lille, France; d Centre Hospitalier Universitaire de Lille, F-59000 Lille, France; e Institut Pasteur de Lille, F-59000 Lille, France; f Sorbonne Université, Inserm, Centre de Recherche Saint-Antoine, CRSA, AP-HP, Saint Antoine Hospital, Gastroenterology department, F-75012 Paris, France; g Paris Center for Microbiome Medicine, Fédération Hospitalo-Universitaire, F-75012 Paris, France; h Univ. Lille, CNRS, Inserm, CHU Lille, UMR9020-U1277 - CANTHER – Cancer Heterogeneity Plasticity and Resistance to Therapies, F-59000 Lille, France; i Sciempathlabo, F-37270 Larçay, France; j Physiogenex, 280 rue de l’Hers, F-31750 Escalquens, France; k Univ. Lille, Inserm, CHU Lille, Institut Pasteur de Lille, U1167 - RID-AGE - Facteurs de risque et déterminants moléculaires des maladies liées au vieillissement, F-59000 Lille, France; l Univ. Lille, Inserm, CHU Lille, UMR-S1172, EGID and DISTALZ, F-59000 Lille, France; m Institut National de la Recherche Agronomique (INRAE), UMR1319 Micalis & AgroParisTech, F-78350 Jouy en Josas, France

**Keywords:** SARS-CoV-2, covid-19, hamsters, gut microbiota, markers of disease severity

## Abstract

Mounting evidence suggests that the gut-to-lung axis is critical during respiratory viral infections. We herein hypothesized that disruption of gut homeostasis during severe acute respiratory syndrome coronavirus 2 (SARS-CoV-2) infection may associate with early disease outcomes. To address this question, we took advantage of the Syrian hamster model. Our data confirmed that this model recapitulates some hallmark features of the human disease in the lungs. We further showed that SARS-CoV-2 infection associated with mild intestinal inflammation, relative alteration in intestinal barrier property and liver inflammation and altered lipid metabolism. These changes occurred concomitantly with an alteration of the gut microbiota composition over the course of infection, notably characterized by a higher relative abundance of deleterious bacterial taxa such as Enterobacteriaceae and Desulfovibrionaceae. Conversely, several members of the Ruminococcaceae and Lachnospiraceae families, including bacteria known to produce the fermentative products short-chain fatty acids (SCFAs), had a reduced relative proportion compared to non-infected controls. Accordingly, infection led to a transient decrease in systemic SCFA amounts. SCFA supplementation during infection had no effect on clinical and inflammatory parameters. Lastly, a strong correlation between some gut microbiota taxa and clinical and inflammation indices of SARS-CoV-2 infection severity was evidenced. Collectively, alteration of the gut microbiota correlates with disease severity in hamsters making this experimental model valuable for the design of interventional, gut microbiota-targeted, approaches for the control of COVID-19.

**Abbreviations:** SARS-CoV-2, severe acute respiratory syndrome coronavirus 2; COVID-19, coronavirus disease 2019; SCFAs, short-chain fatty acids; dpi, day post-infection; RT-PCR, reverse transcription polymerase chain reaction; IL, interleukin. ACE2, angiotensin converting enzyme 2; TMPRSS2, transmembrane serine protease 2.

## Introduction

Severe acute respiratory syndrome coronavirus 2 (SARS-CoV-2), the aetiologic agent of coronavirus disease (COVID)-19, is the greatest global public health concern at present with over 200 million people infected (> 4 million deaths). Although most infected individuals are asymptomatic or mildly symptomatic, up to 5% of patients develop severe disease requiring intensive care. This rate is more important in the elderly and in individuals with co-morbidities.^1^^,2^ COVID-19 has emerged as a multifaceted, multi-system and multi-organ disorder ranging from nonspecific flu-like symptoms, to pneumonia, acute respiratory distress syndrome, multiple organ failure and death.^3^ The gastrointestinal tract hosts a highly diverse and dynamic microbial ecosystem, composed mostly of anaerobic bacteria that is commonly referred to as the gut microbiota. The tightly regulated microbiota-host interplay influences many physiological functions such as digestion, metabolism, mucosal barrier integrity, organ functions and immune homeostasis.^4,5^ In particular, mounting evidence suggests the intricate relationship between the gut microbiome and pulmonary immunity, including in the context of respiratory infections (for reviews,^6–9^). Prior findings have demonstrated that the gut microbiota is altered during respiratory viral infections such as influenza and that dysbiosis might impact on disease outcomes.^8,10–14^ Changes in the composition of the gut microbiota in patients experiencing COVID-19 have been reported.^15–20^ Patients with severe COVID-19 had significant alterations in fecal microbiomes characterized by enrichment of pathogenic bacteria and depletion of beneficial commensals. Although these clinical studies are informative and have identified bacterial taxa associated with SARS-CoV-2 infectivity and disease severity.^15,18–20^ they also have various limitations. Firstly, substantial inter-individual differences and demographic variables necessitate large cohorts to firmly conclude. Secondly, the disease phase at the time of sample collection was not always known and samples were not collected at baseline, before infection. Thirdly, the gut microbiota’s composition is influenced by clinical management. Confounding factors, including medication (e.g. antibiotics) and the use of oxygen at high doses, might complicate the interpretation. It is therefore essential to develop complementary approaches and use experimental models to assess the consequences of SARS-CoV-2 infection on the gut microbiota composition and functionality. We have recently used a nonhuman primate model (the macaque) to address the question. This system recapitulates mild COVID-19 symptoms that have been observed in the majority of human cases. We reported temporal and moderate changes in gut microbiota composition and metabolic output in this model.^21^ For instance the production of the fermentation products short-chain fatty acids (SCFAs), which are instrumental in the control of immune and inflammatory responses,^22,23^ was decreased. The analysis of the gut microbiota composition in an experimental system that mimics severe COVID-19 might be instrumental to better understand the role of the gut-to-lung axis in disease severity. Transgenic mouse expressing human angiotensin converting enzyme 2 (ACE2), the receptor for SARS-CoV-2,^24^ under the control of the epithelial cell cytokeratin-18 promoter, represents a model of severe COVID-19.^25,26^ However, the artificial expression of human ACE2 does not faithfully recapitulate human ACE2 expression and animals suffer from severe SARS-CoV-2-induced encephalitis, a cause of death not observed in humans. In this experimental system, Cao and colleagues recently reported alterations in the composition of the gut microbiota during SARS-CoV-2 infection.^19^ In the present study, we took advantage of a Syrian hamster model recently described to simulate the clinical and pathological manifestations of COVID-19.^27, 27–33^ In this model, SARS-CoV-2 infection led to a progressive alteration of the gut microbiota composition characterized by a higher relative abundance of deleterious bacterial taxa such as Enterobacteriaceae and Desulfovibrionaceae, a reduced relative abundance of SCFA producers (Ruminococcaceae and Lachnospiraceae) and a diminished concentration of SCFAs in blood. However, SCFA supplementation had no effect on clinical parameters in this experimental model of COVID-19. Lastly, several bacterial taxa correlated with clinical and inflammation indices of SARS-CoV-2 infection severity. Collectively, SARS-CoV-2 infection altered the gut-to-lung axis in hamsters suggesting that this experimental model may be instrumental for interventional approaches that aim to target the gut microbiota for a better control of COVID-19.

## Results

### Sublethal SARS-CoV-2 infection causes severe lung disease in hamsters

**Figure 1.**
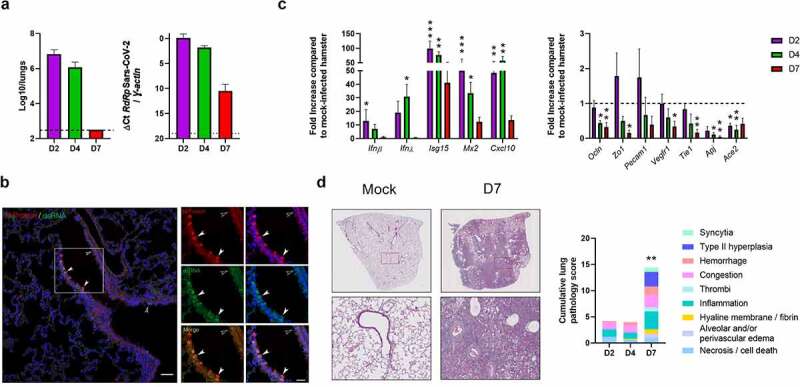
**Establishment of a sublethal model of SARS-CoV-2 infection in hamsters**. Hamsters were inoculated with 2 × 10^4^ tissue culture infectious dose 50 (TCID_50_) of the clinical SARS-CoV-2 isolate hCoV-19/France/lDF0372/2020. (a) Determination of infectious viral loads in the lungs on day 2 (D2), day 4 (D4) and day 7 (D7) post-infection (dpi). Data are expressed as the number of infectious virus particles per lung (*left* panel). Quantification of viral RNA-dependent RNA polymerase (RdRp) transcript levels in the whole lungs was quantified by RT-PCR (*right* panel). Data are expressed as delta Ct values. The dashed line represents the detection threshold. Results are expressed as the mean ± SD. (b) Co-staining for viral N protein (red) and dsRNA (green) in infected lung tissues using immunofluorescence assay (4 dpi). Arrows point to double-positive cells. Nuclei are stained with DAPI (blue). (c) mRNA copy numbers of genes were quantified by RT-PCR (lung). Data are expressed as mean fold increase ± SD over average gene expression in mock-treated animals. (d) Histopathological examination of lung sections of mock-infected and infected hamsters. *Left* panels, Representative images of lungs from mock-infected and SARS-CoV-2-infected hamsters (hematoxylin and eosin staining) in *left* panels. *Lower left* panels, enlarged views of the area circled in red in *upper left* panels. *Right* panel, Blinded sections were scored for levels of pathological severity. To evaluate comprehensive histological changes, lung tissue sections were scored based on criteria indicated in the *right* panel. The following scoring system was used: 0, no pathological change; 1, affected area (≤10%); 2, affected area (<50%, >10%); 3, affected area (≥50%). The average sum of different parameters is shown. (**a-d**), A representative experiment out of two is shown (n = 5/time point) Significant differences were determined using the Kruskal–Wallis ANOVA test (*P < 0.05; ** *P* < 0.01; *** *P* < 0.001)

Male hamsters were infected intranasally with a sublethal dose of a clinical SARS-CoV-2 isolate. Animals experienced body weight loss as early as day 2 post-infection (dpi) (Figure S1a). At 4 dpi, animals began to show ruffled fur and signs of respiratory disease including labored breathing. Weight loss peaked at 7 dpi (average of 17% of initial weight) and from day 7 onward, infected animals started to recover. Infectious virus load in lung homogenates peaked at 2–4 dpi (Figure 1a, *left panel*). At 7 dpi, no virus was detected in the lungs. These data were confirmed by quantification of viral RNA-dependent RNA polymerase levels by quantitative reverse transcription polymerase chain reaction (RT-PCR) (Figure 1a, *right panel*). Trace amounts of viral RNA were detected at 7 dpi. Of note, infectious viruses were not detected in the intestine, liver and blood (not shown). Immunofluorescence assays indicated a focal distribution of the virus in bronchial and bronchiolar epithelia and to a lesser extent in alveoli (pneumocytes and alveolar macrophages) at 4 dpi (Figure 1b and not shown). These data were confirmed by immunohistochemistry using an antibody against the spike protein (Figure S2).

Analyses of gene expression in lungs indicated that infection induced a rapid and transient expression of transcripts encoding interferon (IFN)-β and IFN-λ and a more sustained expression of IFN stimulated genes (ISGs) such as *Isg15, Mx2* (interferon-induced GTP-binding protein), *Cxcl10* (C-X-C motif chemokine ligand 10), *Stat1* (signal transducer and activator of transcription 1), and *Ifi27* (IFNα inducible protein 27) (Figure 1c, *left panel*, Figure S1b). Transcripts encoding cytokines and chemokines such as IL-1β (interleukin-1β), IL-18, IL-6, IFN-γ, IL-10, CCL2 (C-C motif chemokine ligand 2), CCL4 and CCL7 were also up-regulated in the lungs during infection (Figure S1b). SARS-CoV-2 infection also led to a strongly reduced expression of epithelial genes, including *Ocln* (occludin) and *Zo1* (zonula occludens 1), and endothelial genes such as *Pecam1* (platelet endothelial cell adhesion molecule 1), *Vegfr1* (vascular endothelial growth factor receptor 1), *Tie1* (tyrosine kinase with immunoglobulin like and EGF like domains 1) and *Apj* (apelin receptor) particularly at 7 dpi (Figure 1c, *right panel*). SARS-CoV-2 infection also associated with a rapid (2 dpi) and sustained (up to 7 dpi) reduced expression of transcripts encoding Ace 2 (Figure 1c, *right panel*). In line with the finding on transcript levels, Ace 2 protein expression in lung tissue was lowered during infection (Figure S3). Regarding other cellular compounds involved in human SARS-CoV-2 infection, transmembrane serine protease 2 (TMPRSS2)^34^ and neuropilin-1^35^ had also a reduced transcript expression during infection (Figure S1c). Regarding lung pathology, animals developed an acute bronchointerstitial pneumonia at 2 dpi with mild to moderate necrotizing bronchiolitis accompanied by congestion, discrete alveolar hemorrhages, and intra-alveolar and interstitial infiltration of neutrophils and macrophages (Figure 1d). More diffuse bronchointerstitial pneumonia was present at 4 dpi in addition to bronchiolar epithelial cell death/necrosis. Alveolar septal congestion, edema, protein rich fluid exudation, and patchy alveolar hemorrhage were more extensive than at 2 dpi. At 7 dpi, more than 75% of lung sections were affected with sub-acute bronchointerstitial pneumonia, which was characterized by extensive and marked hyperplasia of bronchiolar epithelial cells and type II alveolar pneumocytes, as well as, interstitial, perivascular and intra-alveolar mixed cell inflammation (Figure 1d). Infection was also associated with alveolar collapse and occasional hyaline membranes with some hemorrhages associated with micro-vasculitis and discrete thrombi. The presence of numerous intra-alveolar syncytia pneumocytes was also noticed. Consistent with other studies,^27–33,36^ these findings confirmed that SARS-CoV-2 causes severe pulmonary disease in Syrian hamsters following intranasal exposure.

### Sublethal SARS-CoV-2 infection associates with gut and liver disorders

**Figure 2.**
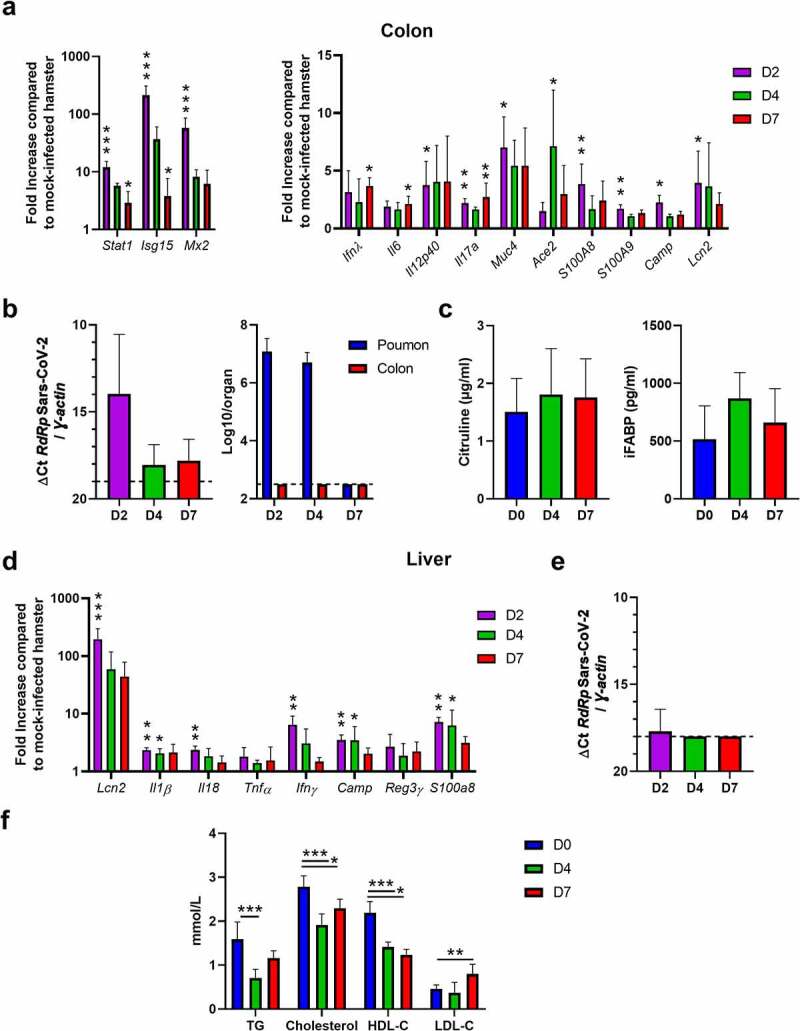
**Analysis of gut disorders during SARS-CoV-2 infection**. Hamsters were infected as described in Figure 1. (a) The colon of mock-infected and SARS-CoV-2-infected hamsters were collected at 2 (D2), 4 (D4) and 7 (D7) dpi. mRNA copy numbers of genes were quantified by RT-PCR. Data are expressed as fold increase over average gene expression in mock-treated animals. (b) *Left panel*, SARS-CoV-2 RNA in the colon was quantified by quantitative RT-PCR. Data are expressed as delta Ct values. *Right panel*, Determination of infectious viral loads in the colon. The number of infectious virus in the lung is depicted for comparison. Data are expressed as the number of infectious virus particles per organ. The dashed line represents the detection threshold. (c) Citrulin (*left* panel) and intestinal fatty-acid binding protein (iFABP) (*right* panel) concentrations in the blood of mock-infected and SARS-CoV-2-infected hamsters. (d) The livers of mock-infected and SARS-CoV-2-infected hamsters were collected at 2, 4 and 7 dpi. mRNA copy numbers of genes were quantified by RT-PCR. (e) Quantification of viral RNA-dependent RNA polymerase (RdRp) transcript levels in the liver was quantified by RT-PCR. Data are expressed as delta Ct values. The dashed line represents the detection threshold. Results are expressed as the mean ± SD. (f) Blood (serum) concentrations of triglycerides (TG), cholesterol, high-density lipoprotein cholesterol (HDL-C), and low-density lipoprotein cholesterol (LDL-C). (a-f) Results are expressed as the mean ± SD. A representative experiment out of two is shown (n = 5 to 8/time point). Significant differences were determined using the Kruskal–Wallis ANOVA test (*P < 0.05; ** *P* < 0.01; *** *P* < 0.001)

Clinical evidence suggests intestinal disorders during SARS-CoV-2 infection.^37–41^ Analysis of postmortem COVID-19 patients indicated histopathological evidence of injury as well as presence of virus particles in the gut tissue. In our hamster infectious system, analyses of gene expression in the colon revealed an early and transient enhancement of IFN-λ and ISGs including *Stat1, Isg15*, and *Mx2*, (Figure 2a). This is in line with the presence of viral RNA (but not infectious virus) mostly at 2 dpi (Figure 2b). To a lower extent, genes involved in inflammation such as *Il6, Il12p40* and *Il17a* were also upregulated. Transcripts encoding mucin 4 (*Muc4*) were also upregulated. Unlike the lung tissue, SARS-CoV-2 infection was associated with enhanced colonic gene expression of *Ace2*. Lastly, transcripts encoding the antimicrobial peptides calgranulin A (S100A8), calgranulin B (S100A9), cationic antimicrobial peptide (Camp) and lipocalin 2 (Lcn2, also a marker of inflammation) were also increased in the colon of SARS-CoV-2-infected hamsters (Figure 2a, *right* panel). In contrast to human studies,^37–41^ histological analysis did not reveal intestinal damage and structural remodeling of the epithelium in hamsters (not shown). This is in agreement with other studies,^27,30^ but in contrast with another study.^36^ The reason for this discrepancy is unclear. This might be explained by differences in the virus preparations and the dose used to inoculate animals. In our setting, the blood concentration of citrulline – a marker of the functional enterocyte mass and metabolic activity – was stable during infection (Figure 2c, *left* panel). We then measured the blood concentration of intestinal fatty-acid binding protein (iFABP), a systemic marker associated with gut inflammation and disrupted gut integrity. As depicted in Figure 2c (*right* panel), iFABP concentration was increased at 4 dpi, albeit not significantly (*P* = 0.164). Disruption in the intestinal epithelial barrier can result in the increased portal influx of bacteria or their products into the liver, where they can cause inflammation and injury. RT-PCR analyses revealed the upregulated expression of genes encoding inflammatory markers in the liver, including acute-phase proteins (*Lcn2*), cytokines (*Il1β, Il18, Tnfα, Ifnγ*), and antimicrobial peptides (*Camp*, regenerating family member 3 gamma/*Reg3γ, S100A8*) (Figure 2d). No viral RNA-dependent RNA polymerase transcript levels was detected in the liver by RT-PCR (Figure 2e). Changes in liver functions during infection may alter serum lipid and lipoprotein levels. Indeed, the levels of triglycerides and total cholesterol in blood were significantly reduced during infection (Figure 2f). This was associated with a significant reduction in high-density lipoprotein cholesterol, the latter being negatively associated with COVID-19 severity in humans.^42,43^ In contrast, relative to mock-infected hamsters, low-density lipoprotein cholesterol levels were significantly higher at 7 dpi. Overall, SARS-CoV-2 infection in hamsters associated with a mild intestinal inflammation and a relative alteration in intestinal barrier property that may lead to liver inflammation and lipid metabolism changes.

### Sub-lethal SARS-CoV-2 infection alters the composition of the gut microbiota in hamsters

**Figure 3.**
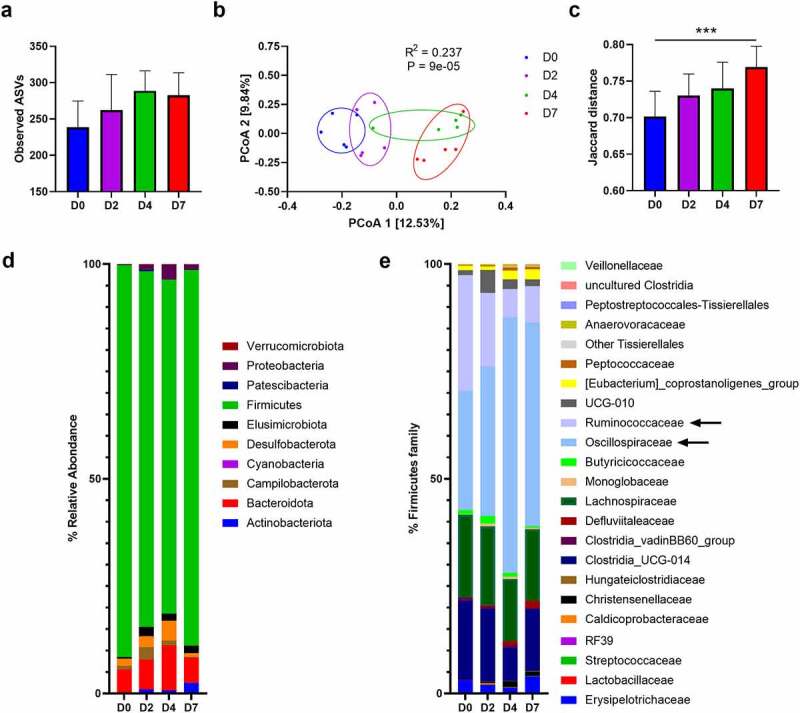
**Changes over time of the gut microbiota’s composition during SARS-CoV-2 infection.** (a) Alpha diversity was estimated by the number of observed Amplicon sequence variants (ASVs). Results are expressed as the mean ± SD. (b) Beta diversity was assessed by principal coordinate analysis (PcoA) of Jaccard index. PC1 and PC2 represent the top two principal coordinates that captured most of the diversity. The fraction of diversity captured by the coordinate is given as a percentage. Each sample is colored according to the time point (day 0 (D0): blue, day 2 (D2): violet, day 4 (D4): green, day 7 (D7):red). (c Jaccard distance between indicated time point and day 0. For day 0, intra group distance is shown, for day 2, 4 and 7, distance to day 0 is shown. Results are expressed as the mean ± SD. (d) and (e) Comparison of the taxonomic composition (phylum in (d) and Firmicutes in (e)) in the gut microbiota of mice in response to SARS-CoV-2 infection. Colored blocks indicate taxa with an average relative abundance. Arrows indicate the most affected families. Significant differences (control *versus* infection) are depicted by * (*P* < 0.05, Kruskal–Wallis ANOVA test). One of two independent experiment is shown (n = 5)

We then turned to analyze the consequences of SARS-CoV-2 infection on the composition of the gut microbiota. To this end, feces samples were collected at 2 dpi, 4 dpi and 7 dpi and were analyzed using 16S rRNA gene amplicon sequencing. Mock-infected animals served as controls. In total, 569,430 sequence reads were analyzed with an average of 28,471 sequence reads per sample (min = 12,980; max = 36,165). Alpha diversity, assessed by the number of observed amplicon sequence variants, Chao1, and Shannon and Simpson indexes, was not statistically modified during the course of SARS-CoV-2 infection although a trend toward an enhancement of abundance and diversity of the bacterial community was observed (Figure 3a and Figure S4a). Beta diversity plots showed clustering by time points (Figure 3b and Figure S4b, *left* panel). Of interest, this analysis revealed a distinct shift in gut microbial composition in SARS-CoV-2-infected hamsters compared to mock-infected hamsters, in particular at 4 dpi and 7 dpi. Analysis of Jaccard (Figure 3c) and Bray Curtis (Figure S4b, *right* panel) distances between day 0 and the other time points showed a drift of the fecal microbiota following infection, peaking at 7 dpi. We next analyzed the composition of the gut microbiota at different taxonomic levels.

**Figure 4.**
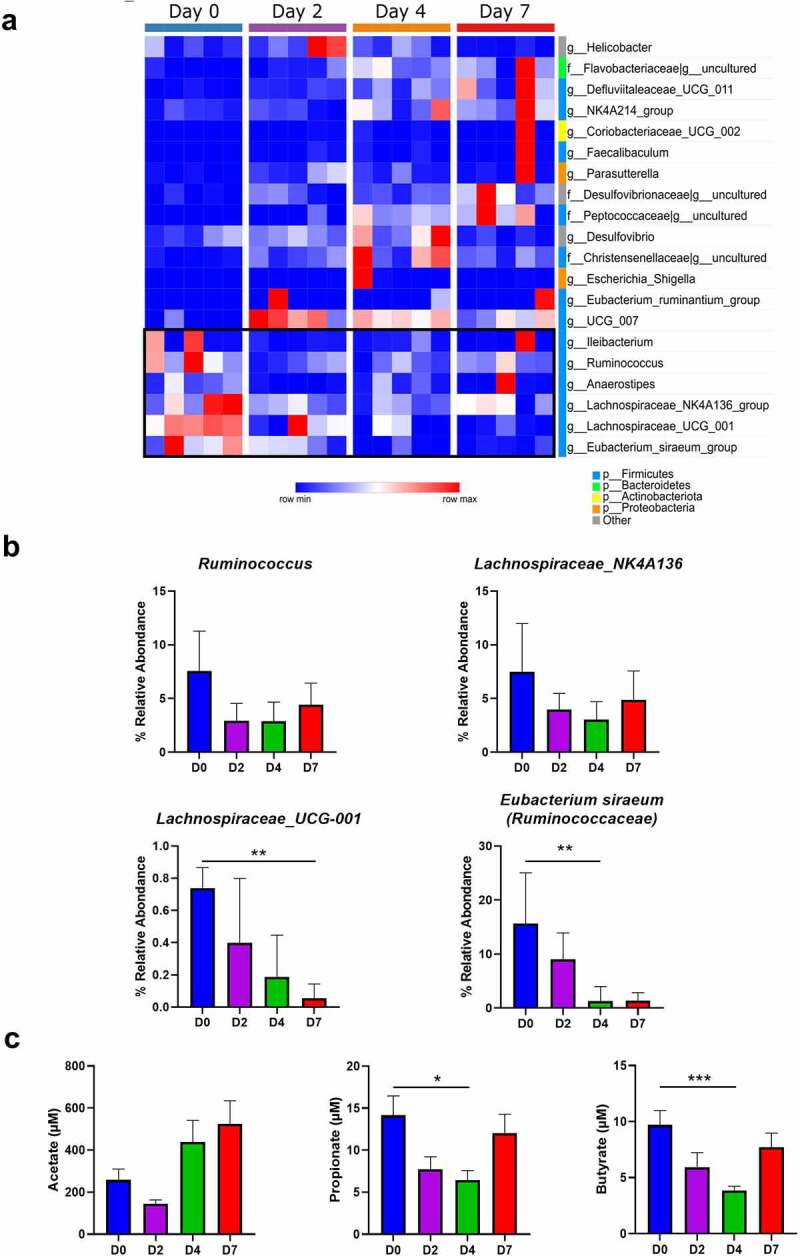
**Changes in bacterial taxa during the course of SARS-CoV-2 infection.** (a) DESeq analysis shows that the representation of the various bacterial taxa changed over the course of the infection. Only taxa with a statistically significant change compared to day 0 are shown (*P* < 0.05; q < 0.15). (b) Relative abundances of some taxa during the course of infection, including those involved in SCFA metabolism, are shown. (n = 5). Results are expressed as the mean ± SD. (c) Blood concentrations in mock-infected and SARS-CoV-2-infected hamsters (n = 6–12, pool of three independent experiments). Data are expressed as the mean ± SD of individual SCFAs. Significant differences were determined using the Kruskal-Wallis ANOVA test (*P < 0.05; **P < 0.01)

At the phylum level, the fecal microbiota of hamsters was dominated by bacteria from the Firmicutes phylum (Figure 3d). Among the other phyla, Bacteroidota (Bacteroidetes), Desulfobacterota, Campilobacterota and, to a lesser extent, Elusimicrobiota were detected. The relative abundance of several phyla changed during infection. Notably, an increased abundance of Bacteroidota, Desulfobacterota, Elusimicrobiota, Proteobacteria, Campilobacterota and Actinobacterioata was noticed, at the expense of Firmicutes. In the latter phylum, the Ruminococcaceae (reduced) and Oscillospiraceae (increased) families were the most affected (Figure 3e). A significant impact of infection was also observed at lower taxonomic levels. Comparison of the microbiota composition between day 0 and the other time points using DESeq2 showed statistically significant differences at the family and genus levels (Figure 4a). Among families differentially affected, the most notable changes were Flavobacteriaceae, Desulfovibrionaceae, Peptoccoccaceae and Christensenellaceae, all enhanced relative abundances. At the genus level, infection was characterized by a marked increase in *Oscillospiraceae_UCG-007* (Clostridia class). To a lower extent, the Gammaproteobacteria class members *Helicobacter, Escherichia-Shigella* (Enterobacteriaceae family) and *Parasuturella* (Sutterellaceae family) had an enhanced relative proportion (Figure 4a). SCFAs, the main gut microbiota metabolites, play important functions in gut homeostasis.^22,44–46^ Of interest, a lower relative abundance of several SCFA-producing Firmicutes (Clostridia class, known to contain important butyrate-producing species) including *Ruminococcus, Lachnospiraceae_NK4A136*, and *Lachnospiraceae_UCG-001* was observed (Figures 4a and 4b). In addition, the acetate producing bacterium *Eubacterium siraeum* (Ruminococcaceae) was less frequent during infection. These data prompted us to quantify SCFA production during SARS-CoV-2 infection. The concentration of acetate (the predominant SCFA), propionate and butyrate in the serum tended to decrease at 2 dpi (Figure 4c). Differences became significant at 4 dpi except for acetate which returned to basal level. At 7 dpi, no significant differences were noted. Overall, SARS-CoV-2 infection in hamsters led to alteration of the gut microbiota composition characterized by an increase of deleterious taxa (Enterobacteriaceae and Desulfovibrionaceae) and a reduction of SCFA producers (Ruminococcaceae and Lachnospiraceae).

### SCFA treatment during SARS-CoV-2 infection failed to improve disease outcomes in hamsters

**Figure 5.**
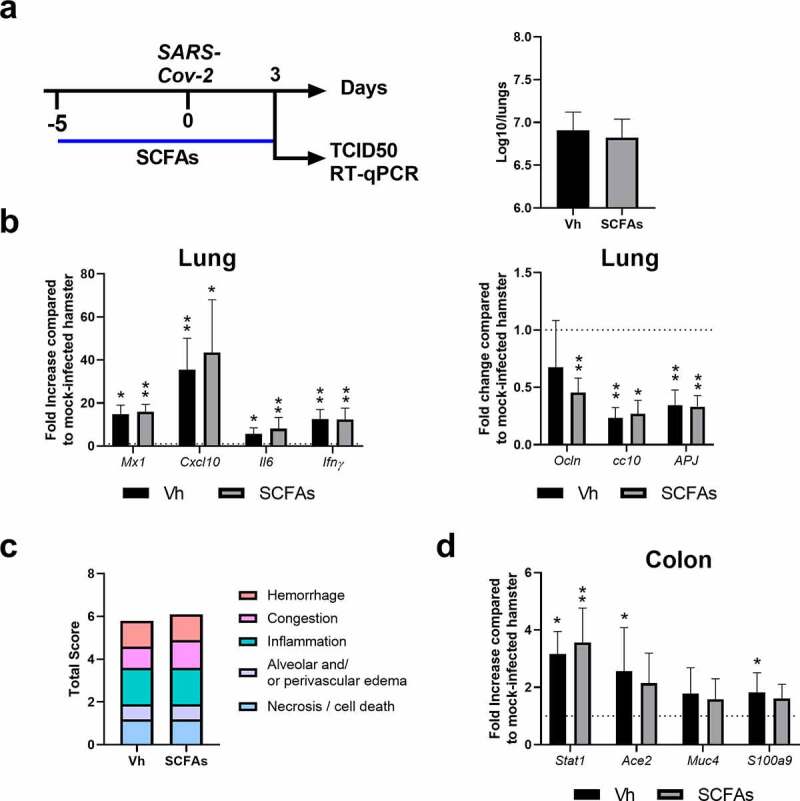
Effect of SCFA supplementation during SARS-CoV-2 infection. (a) *Left* panel, Scheme of the experimental design in which the effects of SCFAs were assessed in SARS-CoV-2-infected hamsters. Animals were treated with acetate (200 mM), propionate (50 mM) and butyrate (20 mM) in drinking water or with vehicle (Vh) five days before infection and during infection. Animals were sacrificed at 3 dpi. a() *Right* panel. Viral load in lungs was assessed by TCID50. (c) mRNA copy numbers of genes were quantified by RT-PCR. Data are expressed as fold increase over average gene expression in mock-treated animals. (d) The average sum of different pathological parameters is shown. (e) Gene expression was assessed in colon (7 dpi). One of two independent experiments is shown (n = 6). Results are expressed as the mean ± SD (b, c and e). Significant differences were determined using the Mann-Whitney U test (*P < 0.05; **P < 0.01)

Previous findings have indicated that SCFAs can improve pathological outcomes during respiratory viral infections including influenza (butyrate) and respiratory syncytial virus (acetate) infections.^47–49^ Mechanisms included reduced tissue damage^47^ and enhanced anti-viral (ISG) response,^48,49^ respectively. In view of this literature and regarding the decrease of SCFAs in our experimental model, we evaluated the impact of SCFA treatment during SARS-CoV-2 infection. To this end, the hamster’s drinking water was supplemented with SCFAs (a mix of acetate, propionate and butyrate) starting 5 days before SARS-CoV-2 infection (Figure 5a, *left* panel). The dose of SCFAs used in the present study was in accordance with that used in previously published studies.^13,14,48,50–52^ SCFA supplementation had no effect on weight loss and viral load in the lungs (Figure S5a and Figure 5a, *right* panel). SCFA treatment also failed to modulate pulmonary transcript levels of ISGs (Mx1), inflammatory cytokines and chemokines (CXCl10, IL-6, IFN-γ) and epithelial (occluding, Clara cell 10-kDa protein/Cc10) and endothelial (APJ) markers (Figure 5b). Analysis of pathological scores showed no difference between vehicle-treated and SCFA-treated animals (Figure 5c). Hence, SCFA supplementation during SARS-CoV-2 infection failed to ameliorate pulmonary disease. SCFA treatment during experimental influenza infection can lower gut disorders.^14^ Analysis of intestinal (Figure 5d) and liver (Figure S5b) gene expression indicated a lack of effect of SCFA treatment. Altogether, SCFA treatment had no impact on the acute phase of COVID-19 in hamsters.

### Changes in gut microbiota composition correlate with COVID-19 severity

**Figure 6.**
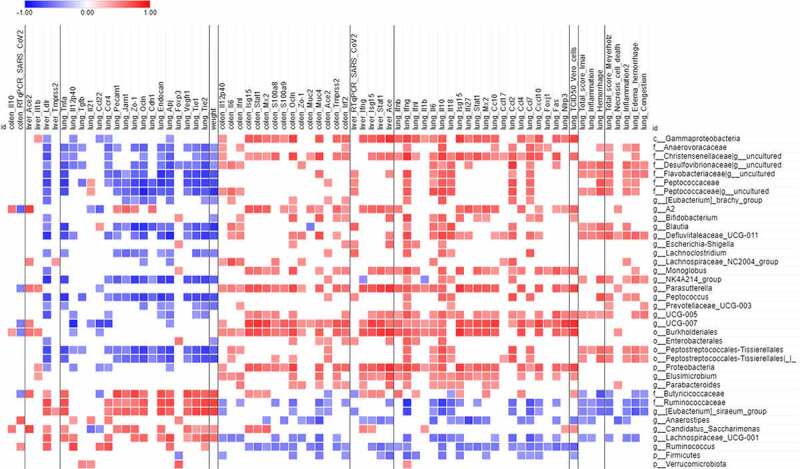
Correlations between bacterial taxa and infection-related variables. Only taxa differentially represented between mock-infected and SARS-CoV-2-infected hamsters (identified with LEfSe) were taken into account. Only significant correlations (p < 0.05 and q < 0.25 after correction for the false discovery rate, using the Benjamini-Hochberg procedure) are shown

The level of inflammatory markers is associated with changes in gut microbiota composition in COVID-19 patients suggesting that dysbiosis might contribute to disease severity via modulation of host immune responses.^17,53^ To investigate this possibility in infected hamsters, we correlated the gut microbiota and clinical and inflammatory indices of SARS-CoV-2 infection severity. We observed a strong correlation between several gut microbiota taxa and clinical and inflammatory indices of SARS-CoV-2 infection severity (Figure 6). For instance, Christensenellaceae, Desulfovibrioaceae, Flavobacteriaceae and Peptococcaceae families positively correlated with lung histological scores and inflammatory cytokines whilst an inverse correlation was seen for Butyricicoccaceae and Ruminococcaceae. These bacterial taxa also positively or negatively correlated with morbidity (weight loss) and ablated pulmonary epithelial and endothelial gene expression. At the genus level, the increased relative abundance of *Blautia, Defluviitaleaeceae_UCG-011, Oscillospiraceae_NK4A214* and *Oscillospiraceae_UCG-005* and the reduced frequencies of *Eubacterium siraeum, Anaerostipes* and *Lachnospiraceae_UCG-001* correlated with pathological and inflammatory indices (Figure 6). It is noteworthy that several taxa positively correlated with viral load and ISG expression in lungs including *Lachnospiraceae*_*A2, Monoglobus, Parasutturella, Oscillospiraceae_UCG-007*, and *Elusimicrobium*. On the other hand, *Anaerostipes* and *Ruminococcus* negatively correlated with viral load and ISG expression in lungs. Collectively, changes in some gut microbiota taxa correlate with clinical and inflammatory indices of SARS-CoV-2 infection severity.

## Discussion

Detailed description of microbial changes that occur during severe COVID-19 might provide important translational information on the understanding of pathological outcomes and might also lead to the discovery of biomarkers of disease severity.^53,54^ The close pathological resemblance with humans makes Syrian hamster a valuable model for studying acute COVID-19. Importantly, the impact of SARS-CoV-2 infection on the gut microbiota composition is still unknown in this model. In the current study, we showed that SARS-CoV-2 infection led to disruption of the gut microbiota composition in hamsters and that several bacterial taxa correlated with markers of disease severity.

The current study confirmed and extended other studies describing the hamster model as an instrumental model for COVID-19.^23,27–33,36^ In this system, the viral load was controlled at 7 dpi and clinical signs including the body weight loss were maximal. Our data emphasized the dramatic lung pathology developing at this time point. Infection and lung pathology associated with an enhanced expression of numerous transcripts encoding ISGs and inflammatory markers and a dramatic reduction of epithelial and endothelial cell markers, in line with the reported epithelial and vascular endothelial damage in severe COVID-19 patients.^55,56^ To a lower extent and in line with several clinical reports, gut disorders also developed during SARS-CoV-2 infection in Syrian hamsters. This was characterized by altered gene expression and by moderate changes in barrier functions, as assessed by enhanced iFABP concentration in blood. The latter was combined with a pro-inflammatory profile in the liver, likely resulting from the portal translocation of bacterial components from the gut. As reported in humans,^42,43^ we also observed altered concentrations of triglycerides and cholesterol-transporting lipoproteins in blood. These alterations might be due to liver dysfunctions. Despite these changes, the intestine and the liver exhibited a normal histological appearance at 7 dpi (not shown). Mild-to-moderate gut disorders during SARS-CoV-2 infection associated with alteration of the gut microbiota composition in hamsters. One may question about the cause(s) of this alteration. Local viral replication might play a role in altered gut microbiota composition. However, in our setting, no infectious virus was detected in the gut. A fecal-oral route of SARS-CoV-2 transmission has been suggested in the hamster model.^30^ It will be interesting to study the impact of oral SARS-CoV-2 infection on the composition and activity of the gut microbiota. The enzymatic activity of Ace 2 is instrumental to maintain the gut’s microbial ecology in part by promoting the synthesis of antimicrobial peptides.^57^ Unlike in the lung compartment (decreased expression), transcripts for Ace2 and antimicrobial peptides increased in the gut during the course of infection, rather eliminating this potential cause. The release of inflammatory cytokines such as type I IFNs^11^ and/or to the reduced food intake^13,58^ might play a part in gut microbiota composition during SARS-CoV-2 infection. Hypoxia (a feature of acute viral respiratory infection), alterations of the enteric nervous system and dysregulated local immune response might also play a role. Further investigation will be necessary to delineate the causes of altered composition of gut microbiota during experimental COVID-19 in hamsters. Regarding the putative role of gut dysbiosis in COVID-19 severity,^15–18^ this key question has a clear application in the clinics.

The gut-lung axis is becoming increasingly important in the context of viral respiratory infections.^8^ Alteration of gut microbiota functions due to acute viral respiratory infections has consequences on disease outcomes such as secondary bacterial infections.^11,13,14,59^ Hence, it is important to detail microbial changes that occur during severe COVID-19. Gut microbiota dysbalance has been described in COVID-19 patients. SARS-CoV-2 infection was associated with a lower relative abundance of bacterial species with known immunomodulatory potential such as *Bifidobacteria, Faecalibacterium prausnitzii* and *Eubacterium rectale*.^17^ Bacteria known to produce the SCFA butyrate such as several genera from the families Ruminococcaceae (i.e. *Ruminococcus bromii and Faecalibacterium prausnitzii*, a major butyrate producer in humans), Lachnospiraceae (*Fusicatenibacter*) and Eubacteriaceae (*Eubacterium hallii)* are also under-represented in COVID-19 patients.^18^ In contrast, SARS-CoV-2 infection is associated with a higher relative abundance of opportunistic bacteria including *Streptococcus infantis, Ruminococcus* (*gnavus* and *torques*), *Bacteroides* (*dorei* and *vulgatus*), *Clostridium, Collinsella aerofaciens*, and *Morganella morganii*, although the extent to which these differences are attributable to concomitant antibiotic treatment in the hospital setting remains unclear.^16–18,20^ Enrichment of these bacteria might lead to local and systemic inflammation. Correlation analyses identified positive association between several bacterial taxa and COVID19 severity.^15,17–19^ It is noteworthy that alterations of the composition of the gut microbiota in SARS-CoV-2-infected hamsters began as early as 2 dpi, more rapidly compared to influenza infection in mice.^13,59^ Beta diversity analysis revealed higher disruption of intestinal communities at 7 dpi. We did notice a higher relative abundance of potentially deleterious taxa such as members of Enterobacteriaceae and Desulfovibrionaceae famillies. At genus level, an enhanced frequency of *Escherichia-Shigella* was observed, in line with a human study.^18^ Other taxa were identified to be strongly affected during SARS-CoV-2 infection in hamsters, including members of the Lachnospiraceae and Ruminococcaceae families. This later observation is in line with Gu and colleagues’s observation made in COVID-19 patients.^18^ The lower relative frequency of these SCFA producers during infection translated into a reduced production of SCFAs early after infection. To a higher extent, a drop of SCFAs was observed during influenza infection in the mouse model.^13,14^ SCFAs are key molecules mediating host-gut microbiota interplay. They exert important regulatory functions in the intestine including a role in antimicrobial responses and mucus secretion, intestinal permeability and activation of the mucosal immune system.^45,46^ They can also distally regulate pulmonary inflammation,^51,60^ including in the context of viral respiratory infections.^47,48,61^ Recently, we showed that the drop of SCFA during influenza participates in secondary bacterial infection, both in lungs (*Streptococcus*) and in the intestine (*Salmonella enterica* serovar Typhimurium).^13,14^ Here, we investigated whether the reduction of SCFAs is relevant for SARS-CoV-2 infection. Clearly, SCFA supplementation did not interfere with SARS-CoV-2 replication in lungs. This is in contrast with another study showing a positive role of acetate on pulmonary viral (respiratory syncytial virus) load.^48^ In contrast, our current data are in line with a lack of butyrate and acetate’s effect on influenza replication *in vivo*.^13,47^ It is also noteworthy that Pascoal and colleagues failed to detect interference effects of SCFAs on SARS-CoV-2 infection *in vitro*.^62^ The role of SCFAs in respiratory viral infections is complex and sometimes controversial. For example, whilst butyrate enhanced viral replication (and reduced ISG expression) *in vitro*,^63^ two independent studies reported that the abundance of butyrate producing bacteria strongly correlated with protection against viral respiratory tract infection.^64,65^ On the basis of these opposite findings, the consequences of butyrate, and in general SCFA, treatment on ISG expression and SARS-CoV-2 replication in our setting could not be predicted. Regarding the sometimes opposing function of SCFAs in viral replication, it would be interesting to test them individually in our setting. SCFA supplementation can lower lung inflammation and injury during respiratory infections. For instance, by inhibiting the activity of histone deacetylases or through binding to the G protein-coupled receptor 41, butyrate reduced pneumonia during *Klebsiella pneumoniae* and influenza virus infection.^47,66,67^ In our COVID-19 model, we failed to evidence positive effects of SCFA supplementation on lung pathology indicating that supplementing SCFAs during SARS-CoV-2 infection had no distal impact on early phase COVID-19 pneumonia. More surprisingly, SCFA treatment did not ameliorate gut inflammation, at least at the gene expression level. Whether SCFA supplementation during SARS-CoV-2 infection impacts on intestinal functions including digestion, metabolism, and mucosal barrier integrity, remains to be investigated in details. This question is of importance regarding the importance of gut disorders in COVID-19 outcomes.^68^ Optimized dose-scheduling to maximize the potential effect of SCFAs and limit potential toxicities is needed in the hamster model to address this question.

Whether changes in intestinal microbiota composition associate with pathological markers was investigated in the current study. Correlation analyses revealed that clinical and inflammatory indices are closely related to several taxa of gut microbiota. Of interest, some of the specific bacterial associations with clinical and inflammatory indices mirrored those observed in humans. For instance, our data supported a relationship between high relative abundance of *Defluviitaleaeceae_UCG-011, Oscillospiraceae_NK4A214* and *Oscillospiraceae_UCG-005* and clinical and inflammatory indices. On the other hand, the reduced frequencies of the SCFA producers *Eubacterium siraeum* and *Lachnospiraceae_UCG-001* correlated with disease severity and inflammation. The reduced frequencies of *Eubacterium* and *Lachnospiraceae species* (respectively *Eubacterium rectale* and *Lachnospiraceae bacterium UGC_5_1_63FAA*) correlate with disease severity in severe COVID-19 patients.^15^ More generally, the negative correlation of SCFA producers (Ruminococcaceae and Lachnospiraceae) and inflammatory markers mirror the situation in humans and nonhuman primates.^15,21^ In our setting, the Gammaproteobacteria class (the genus *Parasutterella)* positively correlated with viral load and inflammatory cytokine expression in lungs. Of note, several studies have reported positive correlation between the relative frequencies of several Gammaproteobacteria members (e.g. *Enterobacter, Escherichia-Shigella, Enterococcus*) and disease severity.^15,18,19^ Further analyses will be required to establish a direct causal relationship between the alterations of gut microbiota and disease severity in this infectious model. Some limitations of this report are worth noting. In our conditions, SARS-CoV-2-infected hamsters had no infectious virus in the intestine and developed a mild intestinal inflammation without injury and overt gut barrier disruption. In severe COVID-19 patients, gut disorders (*e.g*. leaky gut) and altered gut microbiota are likely to be critical in disease severity including cytokine storm and organ dysfonction (for reviews,^54,69,70^). Hence, the hamster model, at least in the conditions used in the current study, is less relevant when referred to gut disorders. It remains to be seen whether comorbidity models such as dyslipidemia-obesity and/or aging recapitulate these features. We have only assessed gut microbiota changes during early COVID-19. As long-lasting effects on microbiota composition have been described in COVID-19 patients,^17,71^ studying later time points will be necessary. In the same line, the potential effect of SCFA supplementation on long COVID-19 needs further investigation. Short-chain fatty acids are just one of many gut-derived metabolite classes that may have physiologic effects during COVID-19, and these include, among others, bile acids, aromatic amino acids, lactate, and succinate^72^ – none of which were studied here. Despite these limitations, our study provides the first description of gut microbiota changes during experimental severe COVID-19 in hamsters. Manipulating the gut microbiota may provide a promising adjuvant therapy to medical procedures in COVID-19 patients.^68,73,74^ Our study does not preclude the use of prebiotics (microbiota-targeted dietary), probiotics (bacteria-based treatment) or gut microbiota derivatives for the management of acute COVID-19. The gut microbiota can indeed generate a plethora of metabolites that can remotely reinforce the antiviral functions of pulmonary immune cells, in part through ISG induction. These immune stimulatory sources include acetate, desaminotyrosine (degradation product of flavonoids) and indole derivatives (produced by tryptophan metabolism).^48,75,76^ Along with these antiviral effects, gut-derived bacteria and components might also reduce acute inflammation and organ failure, impact on long COVID-19 and favor resiliency. This warrants further investigation, in particular in at-risk populations such as the elderlies and comorbid individuals, well-known to have disturbed gut microbiota.

## Materials and methods

### Animals, virus, infection, ethics and biosafety statement

Golden hamsters were purchased from Janvier Laboratory (Le Genest-Saint-Isle, France). Hamsters were fed a standard rodent chow (SAFE A04, Augy, France) and were given water *ad libitium*. The hCoV-19_IPL_France strain of SARS-CoV-2 was used in the current study. Regarding the original Wuhan strain, this strain contains the D614G spike mutation that appeared early in the pandemic and increases viral entry. The SARS-CoV-2 isolate was passed three times on Vero-81 cells (ATCC number CCL-81) expressing the human TMPRSS2. Cells were grown at 37°C with 5% CO_2_ in Dulbecco’s modified eagle medium (DMEM, Gibco) supplemented with 10% heat-inactivated fetal bovine serum (FBS, Eurobio). Sequence data of the virus strain used in the current study are accessible in the National Center for Biotechnology Information (NCBI accession number: MW575140) and Global Initiative on Sharing Avian Influenza Data (GISAID accession number EPI_ISL_940555). The virus was propagated in Vero-E6 cells (ATCC number CRL-1586) expressing TMPRSS2 by inoculation at MOI 0.01. Cell supernatant medium was harvested at 72 h post-infection and stored frozen at −80°C in small aliquots (2 × 10^6^ TCID_50_/mL). All experiments were conducted in a biosafety level 3 laboratory (BSL3) laboratory. For infection, hamsters were anesthetized by intraperitoneal injection of ketamine (100 mg/kg), atropine (0.75 mg/kg) and valium (2.5 mg/kg) and then intranasally infected with 100 µl of DMEM containing (or not, for mock control animals) 2 × 10^4^ TCID_50_ (50% tissue culture infectious dose) of SARS-CoV-2. For tissue collection, animals were euthanized by intraperitoneal injection of euthasol (140 mg/kg). All experiments were performed within the BSL3 of the Institut Pasteur de Lille and complied with current national and institutional regulations and ethical guidelines (Institut Pasteur de Lille/B59-350,009). The protocols were approved by the institutional regional ethical committee ‘Comité d’Ethique en Experimentation Animale (CEEA) 75. The study was authorized by the “Education, Research and Innovation Ministry” under registration number APAFIS#25041-2,020,040,917,227,851 v3.

### Determination of viral load and assessment of gene expression by quantitative RT-PCR

Infectious virus load and viral RNA were determined by using the Reed & Muench tissue culture infectious dose 50 (TCID_50_) assay and quantitative reverse transcription PCR (RT-qPCR), respectively. For titration of live infectious virus, half of right lobes were homogenized in Lysing Matrix D tubes containing 1 ml of PBS using the Mixer Mill MM 400 device (Retsch) (15 min – 15 Hz). After centrifugation at 11,000 rpm for 5 min, the clarified supernatant was harvested for live virus titration. Dilutions of the supernatants were done in DMEM with 1% penicillin/streptomycin and dilutions were transferred to Vero-E6 cells in 96-well plates for TCID50 assay. Briefly, serial 10-fold dilutions of each sample were inoculated in a Vero-E6 cell monolayer in duplicate and cultured in DMEM supplemented with 2% fetal bovine serum (Invitrogen, Waltham, MA) and 1% penicillin/streptomycin and L-glutamine. The plates were observed for cytopathic effects for 5–6 days. Virus titers were expressed as TCID_50_ corresponding to the amount of virus that caused cytopathic effects in 50% of inoculated wells. For viral RNA quantitation in lung tissue, half of the right lobe was homogenized in 1 ml of RA1 buffer (NucleoSpin RNA kit, Macherey Nagel) containing 20 mM of Tris(2-carboxyethyl)phosphine hydrochloride (TCEP). Total RNAs in the tissue homogenate were extracted using the NucleoSpin RNA kit. RNA was reverse-transcribed with the High-Capacity cDNA Archive Kit (Life Technologies, USA). The resulting cDNA was amplified using SYBR Green-based real-time PCR and the QuantStudio™ 12 K Flex Real-Time PCR Systems (Applied Biosystems™, USA) following manufacturers protocol. Relative quantification was performed using the gene coding RNA-dependent RNA polymerase (*RdRp*) and glyceraldehyde 3-phosphate dehydrogenase (*gapdh*). Analyses of gene expression in lungs, colon and liver were performed by classical procedures. Specific primers were designed using Primer Express software (Applied Biosystems, Villebon-sur-Yvette, France) and ordered to Eurofins Scientifics (Ebersberg, Germany). The list of primers is available in Table 1. Relative mRNA levels (2^−ΔΔCt^) were determined by comparing (a) the PCR cycle thresholds (Ct) for the gene of interest and the house keeping gene (ΔCt) and (b) ΔCt values for treated and control groups (ΔΔCt). Data were normalized against expression of the *gapdh* gene and are expressed as a fold-increase over the mean gene expression level in mock-treated mice. Viral load is expressed as viral RNA normalized to *Gapdh* expression level (ΔCt).

**Table 1.** 

*y-actin*	Forward 5ʹ-ACAGAGAGAAGATGACGCAGATAATG-3’	*Il1b*	Forward 5ʹ-GAAGTCAAAACCAAGGTGGAGTTT-3’
	Reverse 5ʹ-GCCTGAATGGCCACGTACA-3’		Reverse 5ʹ-TCTGCTTGAGAGGTGCTGATGT-3’
*RdRp SARS-CoV-2*	Forward 5ʹ-GTGARATGGTCATGTGTGGCGG-3ʹ	*Il18*	Forward 5ʹ-CCGCCATAATCGTCAGGACA-3’
	Reverse 5ʹ-CARATGTTAAASACACTATTAGCATA-3’		Reverse 5ʹ-ACATGGCTTTGGTAGACCACT-3’
*Ifnb*	Forward 5ʹ-ACCCTAAAGGAAGTGCCAG-3’	*Il6*	Forward 5ʹ-CCATGAGGTCTACTCGGCAAA-3’
	Reverse 5ʹ-CCAGCTGCCAGTAATAGCTC-3’		Reverse 5ʹ-GACCACAGTGAATGTCCACAGATC-3’
*Ifnl*	Forward 5ʹ-CCCACCAGATGCAAAGGATT-3’	*Ifng*	Forward 5ʹ-TGTTGCTCTGCCTCACTCAGG-3’
	Reverse 5ʹ-CTTGAGCAGCCACTCTTCTATG-3’		Reverse 5ʹ-AAGACGAGGTCCCCTCCATTC-3’
*Isg15*	Forward 5ʹ-CTGGTGCCCCTGACTAACTC-3’	*Ccl2*	Forward 5ʹ-TGCTAACTTGACGCAAGCTCC-3’
	Reverse 5ʹ-CTGTCATTCCGCACCAGGAT-3’		Reverse 5ʹ-AAGTTCTTGAGTCTGCGGTGG-3’
*Mx2*	Forward 5ʹ-CCAGTAATGTGGACATTGCC-3’	*Ccl4*	Forward 5ʹ-CTCTGCCATGCTTTTGTGCC-3’
	Reverse 5ʹ-CATCAACGACCTTGTCTTCAGTA-3’		Reverse 5ʹ-ATCAGCCCATCTCACCACAG-3’
*Cxcl10*	Forward 5ʹ-TACGTCGGCCTATGGCTACT-3’	*Il12p40*	Forward 5ʹ-AATGCGAGGCAGCAAATTACTC-3’
	Reverse 5ʹ-TTGGGGACTCTTGTCACTGG-3’		Reverse 5ʹ-CTGCTCTTGACGTTGAACTTCAAG-3’
*Ocln*	Forward 5ʹ-GTGGCTTCCACACTTGCTTG-3’	*Il17a*	Forward 5ʹ-GCTGTAGTGAAGGCAGGGTT-3’
	Reverse 5ʹ-GCCACTTCCTGCATAAGGGT-3’		Reverse 5ʹ-GAGGGCCTTCTGGAACTCAC-3’
*Zo1*	Forward 5ʹ-CTCCTGCCGCTCAAAAGGA-3’	*Muc4*	Forward 5ʹ-GGAAAACGAAAACGCCTCCC-3’
	Reverse 5ʹ-CGCCGGAAGTAGCACCATTA-3’		Reverse 5ʹ-AAAATGGATGACCGGACCCC-3’
*Pecam*	Forward 5ʹ-CGCCATGTTGATAGTTGCCG-3’	*S100a8*	Forward 5ʹ-ACTGCTCACGACTGAGTGTC-3’
	Reverse 5ʹ-CTTGAGCTTGGAAGGACGGT-3’		Reverse 5ʹ-GCCAGGCCCACCTTTATCAT-3’
*Vegfr1*	Forward 5ʹ-GCGATACTCGACTTCCCCTG-3’	*S100a9*	Forward 5ʹ-CACGAGTCTAGCAAGGGACA-3’
	Reverse 5ʹ-CACAAGTTCTGCAAACCGGG-3’		Reverse 5ʹ-TGGTTTCTATGCTGCGCTCC-3’
*Tie1*	Forward 5ʹ-CCGGATCCCTATGGCTGTTC-3’	*Camp*	Forward 5ʹ-AACATGGGGTAGTGAAGCGG-3’
	Reverse 5ʹ-TTGATTCGAGGCCTTGTCCC-3’		Reverse 5ʹ-ATCTTTCTTGACCGGCTGGG-3’
*Tie2*	Forward 5ʹ-AGGAGTTTGGGTCTGCAGTG-3’	*Lcn2*	Forward 5ʹ-CCAACCAGCCATTGATCCCT-3’
	Reverse 5ʹ-CGTAGTCAGTCTGAGGCTCC-3’		Reverse 5ʹ-TCACAACGTTGGTCCCTGAG-3’
*Apj*	Forward 5ʹ-CCTCAGCTTCGACCGATACC-3’	*Tnfa*	Forward 5ʹ-TGAGCCATCGTGCCAATG-3’
	Reverse 5ʹ-CCTTGGTGCTGTTTTCCGTG-3’		Reverse 5ʹ-AGCCCGTCTGCTGGTATCAC-3’
*Ace2*	Forward 5ʹ-CTGGGAAAACTCCATGCTG-3’	*Reg3g*	Forward 5ʹ-CCTGTCGCGAAACTCAGGAT-3’
	Reverse 5ʹ-GAACGATCTCTCGCTTCATCT-3’		Reverse 5ʹ-GACAGGTTTGGACTGTGGGA-3’
*Stat1*	Forward 5ʹ-TCCATGCGGTTGAACCCTAC-3’	*Mx1*	Forward 5ʹ-GGTATCGTTACCAGGTGCCC-3’
	Reverse 5ʹ-TGTCAGTGTTCTGTGCTCACTT-3’		Reverse 5ʹ-GGTCTGGAACACTTGGGGAG-3’
*Ifi27*	Forward 5ʹ-TCGTTGCTGCTCCCGTAGTC-3’	*Cc10*	Forward 5ʹ-ACAAGCCCTCTGTGCAATCA-3’
	Reverse 5ʹ-ATGGATCCCGCTGCAATTC-3’		Reverse 5ʹ-GGGGCTGTTATCAGGGAGTG-3’
*Il10*	Forward 5ʹ-GGTTGCCAAACCTTATCAGAAATG-3’	*Ccl7*	Forward 5ʹ-CTCTGCCATGCTTTTGTGCC-3’
	Reverse 5ʹ-TTCACCTGTTCCACAGCCTTG-3’		Reverse 5ʹ-ATCAGCCCATCTCACCACAG-3’
*Tmprss2*	Forward 5ʹ-GGGCTACGAGCTTTATGAAGC-3’	*Nrp1*	Forward 5ʹ-CTGGAAAGAAGGGCGTGTCT-3’
	Reverse 5ʹ-GGACGAACAGGAGTCACTGTG-3’		Reverse 5ʹ-CTTCATATCCGGGGGTGCTC-3’

### Histopathology

Lung tissues were fixed in 4% PBS buffered formaldehyde for 7 days, rinsed in PBS, transferred in ethanol (70%) and then processed into paraffin-embedded tissues blocks. The subcontractor Sciempath Labo (Larçay, France) performed histological processing and analysis. Tissue sections (3 µm) were stained with hematoxylin and eosin (H&E) and whole mount tissues were scanned with a Nanozoomer (Hamatsu) and the morphological changes were assessed by a semi-quantitative score. For the scoring, a dual histopathology scoring system adapted from ^28,77^ was used to assess pulmonary changes in hamsters. A total of nine parameters was qualitatively assessed and ranked with a score from 0 to 4: (1) cellular death/necrosis, (2) alveolar and/or perivascular edema, (3) hyaline membrane or fibrin, (4) inflammation, (5) thrombi, (6) congestion, (7) hemorrhage, (8) type II hyperplasia, and (9) syncytia. For each criteria, a score 0 = absent, 1 = 1–10% of lung section, 2 = 11–25% of lung section, 3 = 26–50% of lung section, and 4 = >50% of lung section affected.

### Immunofluorescence staining and immunohistochemistry

For identification and localization of SARS-CoV-2-N protein and dsRNA in tissues, immunolabeling was performed on deparaffinized and rehydrated tissue sections using rabbit anti-SARS-CoV nucleocapsid (N) protein (Novus Biologicals, Littleton, CO) and mouse anti-dsRNA antibodies (SCICONS J2 Nordic-MUBio, Susteren, The Netherlands). Tissue sections were first treated with antigen unmasking solution (10 mM sodium citrate in TBS) for 30 min at 70°C. Then, sections were rinsed 4 times in 0.1 M PBS pH 7.4 and blocked for 1 hour at room temperature in blocking solution (PBS containing 10% normal donkey serum and 0.3% Triton X-100). Sections were incubated overnight at 4°C with a mix of primary antibodies diluted in blocking solution. Sections were then washed three times in 0.1 M PBS and incubated at room temperature for 1 hour with Alexa Fluor-conjugated secondary antibodies (Molecular Probes, Invitrogen, San Diego, CA) in blocking solution. The sections were rinsed 3 times in 0.1 M PBS. Nuclei were then counterstained by incubating the sections for 1 minute in 4ʹ,6-diamidino-2-phenylindole (Sigma, 1:5000 in TBS). Finally, the sections were incubated with Autofluorescence Eliminator Reagent (Merck-Millipore, Molsheim, France) and mounted with Fluoromount™ (Sigma-Aldrich, Saint-Louis, MO, USA). Images were acquired using an Axio Imager Z2 Apotome microscope (Zeiss, Germany). Immunohistochemistry of hamster tissue sections was performed using anti-ACE2 or anti-SARS-CoV-2 spike protein (Abcam, Cambridge, UK). Sections were incubated with appropriate secondary HRP-conjugated antibody, washed and incubated with VECTASTAIN®Elite ABC-HRP kit (Vector laboratories) following manufacturer’s instructions. Slides were washed three times in PBS and the 3,3ʹ-diaminobenzidine (DAB) chromogen DAB Peroxidase (HRP) Substrate Kit (Vector Laboratories, Burlingame, CA) was added on each slide. Counterstaining was performed using Mayer’s hematoxylin (Merck, Darmstadt, Germany). Finally, slides were mounted with Dako mounting medium. Images were acquired using an Axio Scan.Z1 slide scanner and ZEN (Blue edition) 2012 software (Carl Zeiss, Oberkochen, Germany).

### Gut microbiota analysis

To study the impact of SARS-CoV-2 infection on gut microbiota, hamsters were intranasally infected and their feces were collected at 2 dpi, 4 dpi and 7 dpi. Feces from mock-infected hamsters served as controls. Fecal samples were stored at −80°C until further analyses. Microbial DNA was extracted from 200 mg of fecal samples as previously described.^78^ Following microbial lysis with both mechanical and chemical steps, nucleic acids were precipitated in isopropanol for 10 minutes at room temperature, incubated for 15 minutes on ice and centrifuged for 30 minutes at 15,000 *g* and 4°C. Pellets were resuspended in 112 µl of phosphate buffer and 12 µl of potassium acetate. After RNase treatment and DNA precipitation, nucleic acids were recovered via centrifugation at 15,000 *g* and 4°C for 30 minutes. The DNA pellet was resuspended in 100 µl of TE buffer. The concentration of extracted DNA was determined using on a DNA fluorometric intercalant (SYBR® Green, ThermoFisher Scientific (Waltham, MA). Microbial diversity and composition were determined for each sample by targeting a portion of the ribosomal genes. A 16S rRNA gene fragment comprising V3 and V4 hypervariable regions (16S; 5′-TACGGRAGGCAGCAG-3′ and 5′-CTACCNGGGTATCTAAT-3′) was amplified using an optimized and standardized 16S-amplicon-library preparation protocol (Metabiote, GenoScreen, Lille, France). Briefly, 16S rRNA gene PCR was performed using 5 ng genomic DNA according to the manufacturer’s protocol (Metabiote) using 192 bar-coded primers (Metabiote MiSeq Primers, GenoScreen) at final concentrations of 0.2 μM and an annealing temperature of 50°C for 30 cycles. The PCR products were purified using an Agencourt AMPure XP-PCR Purification system (Beckman Coulter), quantified according to the manufacturer’s protocol, and multiplexed at equal concentrations. Sequencing was performed using a 250-bp paired-end sequencing protocol on an Illumina MiSeq platform (Illumina) at GenoScreen. Positive (artificial bacteria community comprising 17 different bacteria (ABCv2)) and negative (sterile water) control were also included. Following DNA extraction and sequencing, raw paired-end reads were processed in a data curation pipeline that includes a step of removal of low quality reads (Qiime2 2020.6). Remaining sequences were assigned to samples based on barcode matches, and barcode and primer sequences were then trimmed. The sequences were denoized using the DADA2 method, and reads were classified using Silva reference database (version 138). Alpha and beta diversity were computed using the phyloseq package (v1.24.2). Principal Coordinate analyses of the Bray Curtis distance and Jaccard index were performed to assess beta diversity. The number of observed species, Chao1, Shannon and Simpson indexes were calculated using rarefied data (depth = 7,500 sequences/sample) and used to characterize alpha diversity. Raw sequence data are accessible in the National Center for Biotechnology Information (project number PRJNA761913), biosample accession numbers SRX12130168 to SRX12130187. Differential analysis was performed using DESeq2 or the linear discriminant analysis effect size (LEfSe) pipeline. Spearman’s correlations between bacterial taxa and SARS-CoV-2 infection parameters were analyzed. Correlations were considered significant when *P* values < 0.05 with q < 0.15 after correction for the false discovery rate, using the Benjamini-Hochberg procedure.

### Measurement of SCFA concentrations and treatment with SCFAs

Concentrations of SCFAs in blood were determined by ProDigest (Gent, Belgium) after extraction with acetonitrile using GC-2014 gas chromatography with AOC-20i auto injector (Shimadzu, Hertogenbosch, the Netherlands) as described.^79^ To assess the effects of SCFAs on SARS-CoV-2 infection, hamsters infected with SARS-CoV-2 were treated (drinking water) five days before infection and during infection with a combination of sodium acetate (200 mM), propionate (50 mM) and butyrate (20 mM) (Sigma Aldrich).

### Quantification of blood factors

Quantification of triglycerides (TG), high-density lipoprotein (HDL) cholesterol, low-density lipoprotein (LDL) cholesterol in serum was performed using a Horiba Pentra 400 machine and related Pentra assay kits (Horiba France SAS, Longjumeau, France).

## Statistical analyses

Results are expressed as the mean ± standard deviation (SD) unless otherwise stated. All statistical analyses were performed using GraphPad Prism v6 software. A Mann-Whitney *U* test was used to compare two groups unless otherwise stated. Comparisons of more than two groups with each other were analyzed with the One-way ANOVA Kruskal-Wallis test (nonparametric), followed by the Dunn’s posttest. P < 0.05; **, P < 0.01; ***, P < 0.001.

## Supplementary Material

Supplemental Material

## Data Availability

Please contact author for data requests
