# Supplementary figures and images for: Alteration of the gut microbiota following SARS-CoV-2 infection correlates with disease severity in hamsters
Source: Gut Microbes. 2021 Dec 29;14(1):2018900. doi: 10.1080/19490976.2021.2018900 (PMC8726722; doi:10.1080/19490976.2021.2018900)

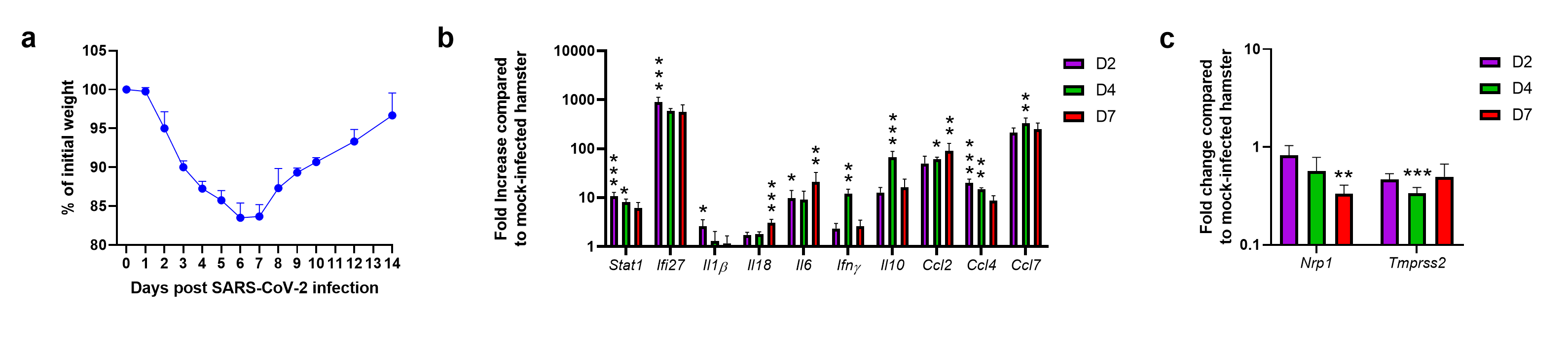

Supplement: Supplemental Material [file KGMI_A_2018900_SM6587.zip › supplementary/Figure Sup1 revised.tif]

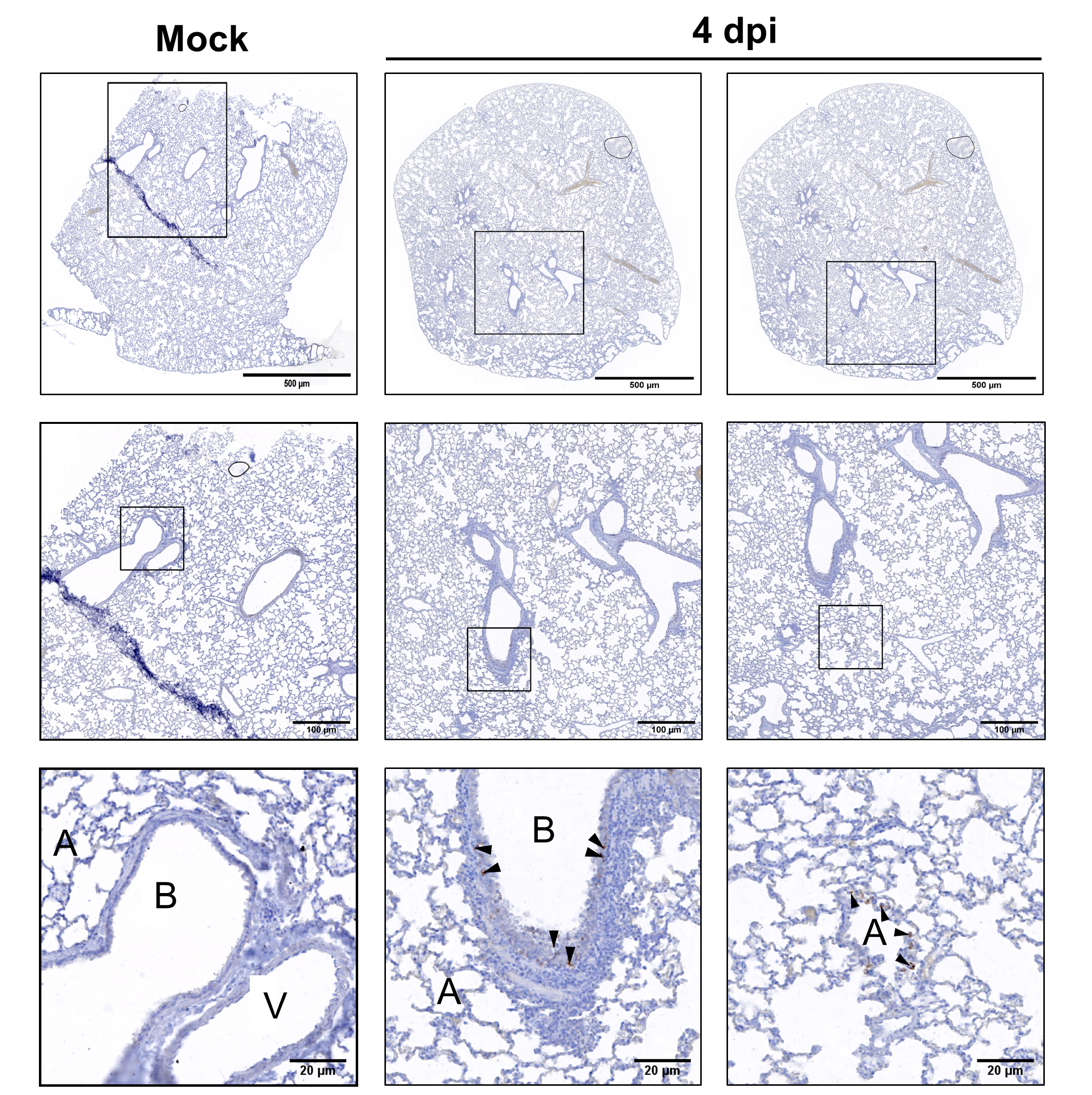

Supplement: Supplemental Material [file KGMI_A_2018900_SM6587.zip › supplementary/Figure Sup2.tif]

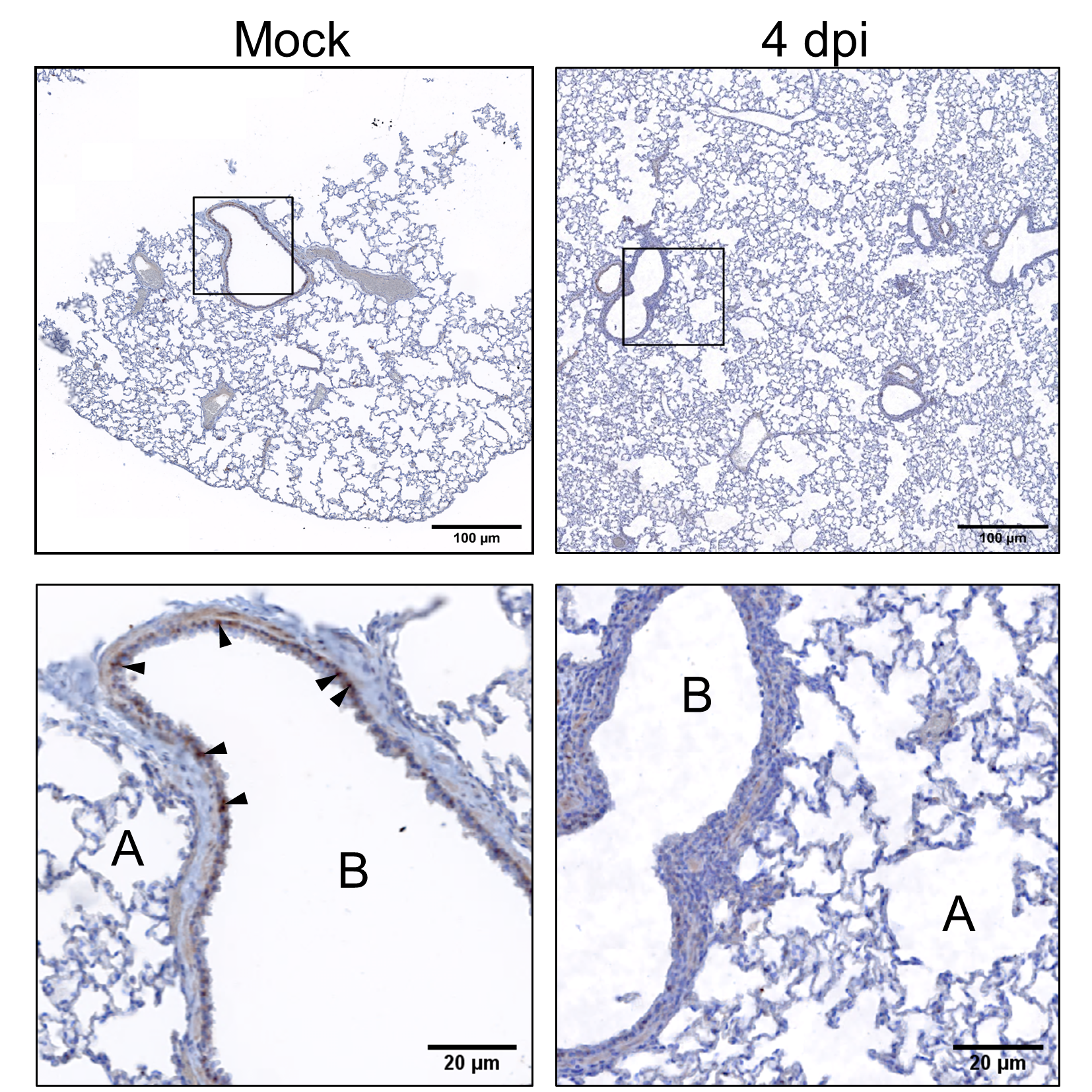

Supplement: Supplemental Material [file KGMI_A_2018900_SM6587.zip › supplementary/Figure Sup3.tif]

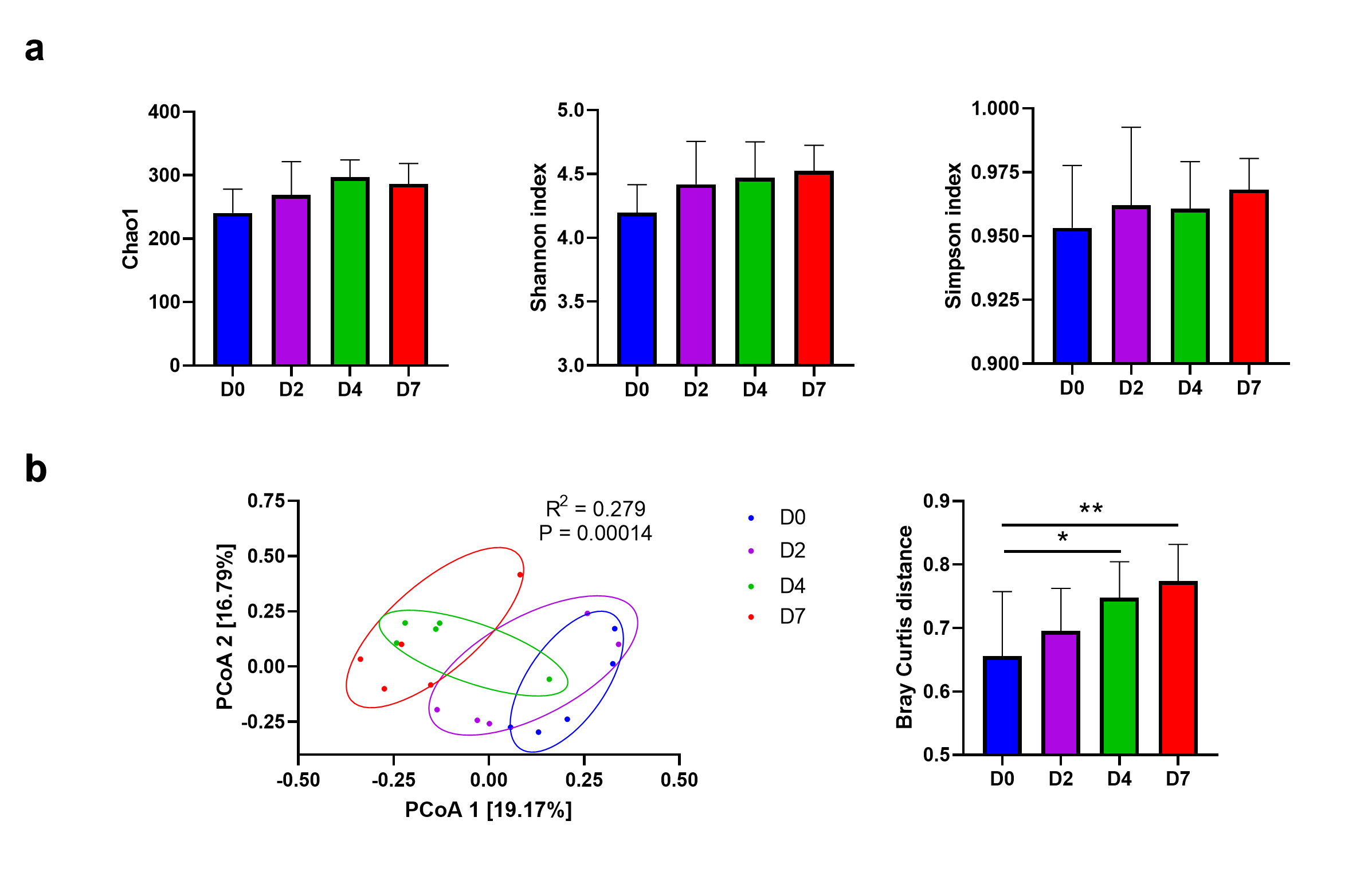

Supplement: Supplemental Material [file KGMI_A_2018900_SM6587.zip › supplementary/Figure Sup4.tif]

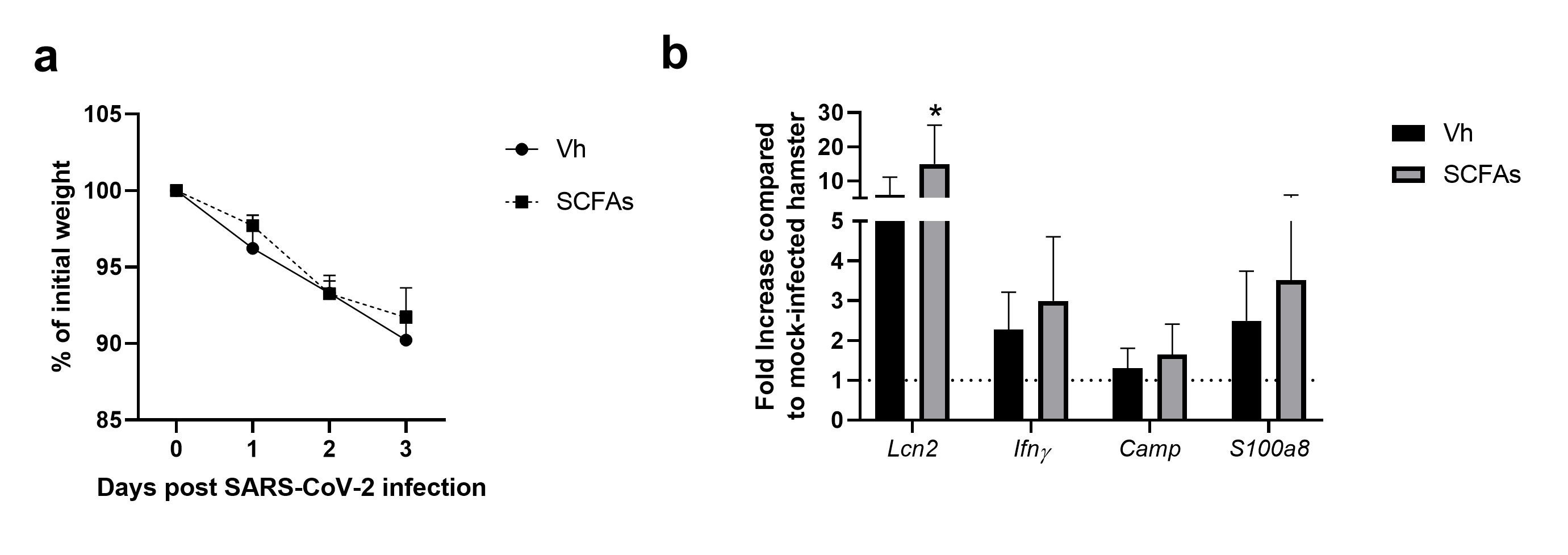

Supplement: Supplemental Material [file KGMI_A_2018900_SM6587.zip › supplementary/Figure Sup5.tif]
